# Effects of Low-Load Blood Flow Restriction Resistance Training on Muscle Strength and Hypertrophy Compared with Traditional Resistance Training in Healthy Adults Older Than 60 Years: Systematic Review and Meta-Analysis

**DOI:** 10.3390/jcm11247389

**Published:** 2022-12-13

**Authors:** Raúl Fabero-Garrido, Miguel Gragera-Vela, Tamara del Corral, Juan Izquierdo-García, Gustavo Plaza-Manzano, Ibai López-de-Uralde-Villanueva

**Affiliations:** 1 Department of Radiology, Rehabilitation and Physiotherapy, Faculty of Nursing, Physiotherapy and Podiatry, Complutense University of Madrid, 28040 Madrid, Spain; 2 Department of Radiology, Rehabilitation and Physiotherapy, Faculty of Nursing, Physiotherapy and Podiatry, Complutense University of Madrid, Instituto de Investigación Sanitaria del Hospital Clínico San Carlos (IdISSC), 28040 Madrid, Spain; 3 Multidisciplinary Cardiac Rehabilitation Unit, University Hospital 12 de Octubre, 28041 Madrid, Spain

**Keywords:** blood flow restriction therapy, aged, healthy people programs, human physical conditioning, musculoskeletal physiological phenomena, review

## Abstract

Background: There is clinical interest in determining the effects of low-load blood flow restriction (LL-BFR) resistance training on muscle strength and hypertrophy compared with traditional high- and low-load (HL and LL) resistance training in healthy older adults and the influence of LL-BFR training cuff-pressure on these outcomes. Methods: A search was performed on the MEDLINE, PEDro, CINHAL, Web of Science, Science Direct, Scopus, and CENTRAL databases. Results: The analysis included 14 studies. HL resistance training produces a small increase in muscle strength (eight studies; SMD, −0.23 [−0.41; −0.05]) but not in muscle hypertrophy (six studies; (SMD, 0.08 [−0.22; 0.38]) when compared with LL-BFR resistance training. Compared with traditional LL resistance training, LL-BFR resistance training produces small–moderate increases in muscle strength (seven studies; SMD, 0.44 [0.28; 0.60]) and hypertrophy (two studies; SMD, 0.51 [0.06; 0.96]). There were greater improvements in muscle strength when higher cuff pressures were applied versus traditional LL resistance training but not versus HL resistance training. Conclusions: LL-BFR resistance training results in lower muscle strength gains than HL resistance training and greater than traditional LL resistance training in healthy adults older than 60 years. LL-BFR resistance training promotes a similar muscle hypertrophy to HL resistance training but is greater than that of traditional LL resistance training. Applying cuff pressures above the limb occlusion pressure could enhance the increases in muscle strength compared with traditional LL resistance training.

## 1. Introduction

Life expectancy has been increasing significantly worldwide in recent years, with an expected doubling of the population older than 60 years predicted to happen by 2050 [1]. The aging process tends to reduce muscle strength and mass, which profoundly affects the functionality and disability [2], resulting in a higher number of falls, hospital admissions, frailty, and mortality [3]. Furthermore, the sedentary behavior that predominates in older age results in the premature onset of ill health and frailty [4] and has detrimental effects on the cardiometabolic markers associated with cardiovascular disease [5].

According to the most recent systematic reviews, resistance training should be considered as the primary non-pharmacological therapy to manage the loss of muscle mass and strength in older adults [6,7]. Resistance training delays or reverses sarcopenia increases the skeletal muscle mass, strength and power, enhances mobility, physical functioning, performance in activities of daily living and psychosocial well-being, preserves independence, and reduces the risk of falls [7,8]. The intensity of the training appears to be a critical variable, with greater effects on the strength gains in older adults from high-load (HL) resistance training compared with moderate- and low-load (LL) resistance training [9,10,11], even in frail older adults [12]. However, HL exercises might be contraindicated for older adults with specific pathological conditions and those unable to lift a sufficient weight to induce hypertrophy [13].

In recent years, a promising new resistance training modality has emerged: blood flow restriction (BFR), which uses a pneumatic cuff to partially or totally occlude the arterial and venous blood flow during exercise [14]. Among the advantages of this type of training is the use of low-intensity training (20–30% of one repetition maximum [1RM]), called LL resistance training with BFR (LL-BFR), which generates physiological stress and an activation of the anabolic pathways that increase muscle size and strength similar to that of traditional HL resistance training (≥70% 1RM) [14,15,16].

Several systematic reviews [17,18,19] have already explored the muscle strength and mass benefits of LL-BFR compared with traditional HL and LL resistance training in older adults; however, the reviews have several limitations that need to be carefully addressed. The reviews included studies with highly heterogeneous samples that did not distinguish between healthy participants and older adult participants with the disease [18,19] and included individuals younger than 60 years (50–60 years) [17,18], who should not be considered as older adults. The reviews’ conclusions might therefore be suspect and require a careful interpretation before extrapolating them to older adults without pathological conditions.

Previous reviews have highlighted the large variability in BFR training protocols, especially regarding the cuff pressure, requiring further study. Several studies on healthy young adults have suggested that high cuff pressures are uncomfortable [20,21] and do not appear to result in enhanced muscle adaptations [22]. Therefore, there is a gap in the knowledge concerning the optimal cuff pressure for the maximum adaptation of aging human skeletal muscle. Identifying the resistance training strategies that offer the same benefits as high-intensity training could counteract the functional decline occurring with progressive age. LL-BFR training might therefore be a more feasible approach to improving the muscle’s strength and function over an individual’s lifetime.

The present systematic review and meta-analysis aimed to provide an update on the effects of LL-BFR resistance training on muscle strength and hypertrophy compared with traditional HL and LL resistance training in healthy adults older than 60 years. As a secondary objective, we analyzed the influence of the cuff pressure of LL-BFR training on muscle strength and hypertrophy in this population.

## 2. Materials and Methods

This systematic review and meta-analysis followed the criteria of the preferred reporting items for systematic reviews and meta-analysis (PRISMA) guidelines [23] and was registered in PROSPERO (CRD42022323396).

### 2.1. Study Selection Criteria

The inclusion and exclusion of the reviewed studies relied on the clinical and methodological aspects based on the PICO (Population-Intervention-Comparison-Outcome of interest) strategy [24].

- Population: The study participants had to be healthy and older than 60 years, in accordance with the currently accepted thresholds for senescence [25], with no gender limitation. No participant was included who had engaged in structured training in the previous 3 months. We excluded the data from participants who had any disorders.
- Intervention and comparison: The included studies had to compare a BFR resistance training against (1) an active HL control group in which resistance training was performed without BFR at high intensities (≥70% of 1RM) or (2) an active LL control group in which resistance training was performed at 20–40% of 1RM without BFR. The minimum training period was 3 weeks, given that this is the minimum period for physiological adaptations to occur with BFR training [26]. BFR interventions on the lower and/or upper limb strength were included.
- Outcomes: The outcomes of interest were the muscle strength and hypertrophy; thus, all the included studies had to assess at least one of these factors. Muscle strength was assessed using maximal dynamic strength by 1RM tests (found directly or reliably estimated from 10RM [27]), measuring the maximal isometric and isokinetic strength. These measures have been found to be valid and reliable in the evaluation of muscle strength in older adults [28,29,30]. The muscle hypertrophy was evaluated by the muscle cross-sectional area, estimated muscle mass, muscle thickness, and body perimeters, all having proven to be valid and reliable methods [31,32,33,34]. These measurements could be performed anywhere on both the upper and lower limb musculature.
- Study design: Only randomized controlled trials and crossover trials were included. Articles were included if they were published in English, Spanish, or Portuguese.

### 2.2. Search Strategy

The search strategy was performed following the guidelines of Russell-Rose et al. [35]. The searches were conducted in the MEDLINE, PEDro, CINHAL, Web of Science, Science Direct, Scopus, and CENTRAL electronic databases, with no date restrictions, up to 15 March 2022. The search string was created with three sections: the first encompassed synonyms for the MeSH term “aged” (e.g., elderly, older adults, or senior); the second was composed of synonyms for the MeSH term “blood flow restriction therapy” (e.g., restriction training, vascular occlusion, or KAATSU); and the third included a high-quality filter of a randomized controlled trail. To ensure the inclusion of a study with at least one search term within a section, all the synonyms were connected with the “OR” boolean operator, while the sections were connected with the “AND” boolean operator. The search string was adapted to each database, according to the data in File S1. To detect the additional relevant studies, the references of previously published systematic reviews in this field were reviewed. If additional information from the studies was needed, the authors were contacted by e-mail. Two independent reviewers conducted the search using the same methodology (RFG and MGV), and any discrepancies were resolved with the intervention of a third reviewer (ILUV).

### 2.3. Selection Criteria and Data Extraction

In the first phase, two independent reviewers (RFG and MGV) screened the titles, abstracts, and keywords of the studies following the Cochrane recommendations [36]. In the second phase, full-text copies of peer-reviewed relevant papers were reviewed and checked as to whether they met the inclusion criteria and to identify and record the reasons for excluding ineligible studies. A third reviewer (ILUV) was consulted in case of a disagreement. The relevant data were extracted for each included study (RFG and MGV).

### 2.4. Methodological Quality and Risk of Bias Assessment

The PEDro scale was employed to assess the quality of the included trials because it is a reliable method for assessing the quality of randomized controlled trials [37,38]. The PEDro scale consists of 11 items, with a maximum score of 10 points. The total score for each study was stratified as follows: poor (<4 points), fair (4–5 points), good (6–8 points), and excellent (9–10 points) [38]. The risk of bias for each included study was assessed in accordance with the Cochrane recommendations using 6 criteria that were individually rated [36]. For each domain, the risk of bias was categorized as high, low, or uncertain, and the reasons were recorded along with a descriptive justification for the judgement. In the “other bias” category, we clarified the specific criteria that could have affected the results.

Two independent trained assessors (RFG and MGV) examined the quality and risk of bias of the selected studies using the same methods, and disagreements were resolved by a consensus or by consulting the third reviewer (ILUV). The inter-rater reliability was determined using the Kappa coefficient: (1) >0.81–1.00 indicated an excellent agreement between the assessors; (2) 0.61–0.80 indicated a good agreement; (3) 0.41–0.60 indicated a moderate agreement; and (4) 0.21–0.40 indicated a poor agreement [39].

### 2.5. Qualitative Analysis

The qualitative analysis was performed according to the Grading of Recommendations, Assessment, Development, and Evaluation (GRADE), following the recommendations of Andrews et al. [40].

### 2.6. Data Analysis

The statistical analysis was performed with RStudio 3.0 software using the ‘metaphor’ and ‘esc’ packages. All the significance tests were conducted at a level of 5%. A meta-analysis was performed only when the data for the analyzed variables were represented in at least 3 studies/comparisons. The data synthesis was categorized by groups according to the degree of limb cuff pressure: the cuff pressure near the limb occlusion pressure versus being greater than the limb occlusion pressure [41].

To increase the accuracy and thus the generalizability of our analyses, multiple comparisons from several studies (e.g., isotonic and isometric force measures) were included in all the analyses [42]. In the pre- and post-intervention, the mean difference and standard deviation (SD) values for the muscle strength and hypertrophy in each study/comparison were used to calculate the standardized mean difference (SMD). The change in the SD was calculated in accordance with the Cochrane recommendations, using the most conservative model (r = 0) [36]. When necessary, the mean scores and SDs were estimated from the graphs.

The summary statistics for all the analyses are presented using forest plots. A random-effects model was employed to determine the overall effect size (SMD). The effect size of the statistical significance of the overall SMD was examined using Hedges’ g and interpreted as follows: (1) a small effect (g = 0.20–0.49); (2) a moderate effect (g = 0.50–0.79); and (3) a large effect (g ≥ 0.80) [43].

The degree of heterogeneity among the studies was estimated using Cochran’s Q statistic test and the inconsistency index (I^2^) [44]. Heterogeneity was considered when the Cochran’s Q statistic test was significant (*p* < 0.1) and/or the I^2^ was >50% [45].

Influential or outlier studies were investigated according to the recommendations of Viechtbauer and Cheung [46]. The robustness of the results obtained in the meta-analysis was assessed with a leave-one-out sensitivity analysis [47].

To detect any publication bias, the funnel plots were visually assessed, with an asymmetric graph considered to indicate the presence of bias. Egger’s regression test was employed to quantitatively assess the publication bias [48,49].

## 3. Results

### 3.1. Study Selection

**Figure 1:**
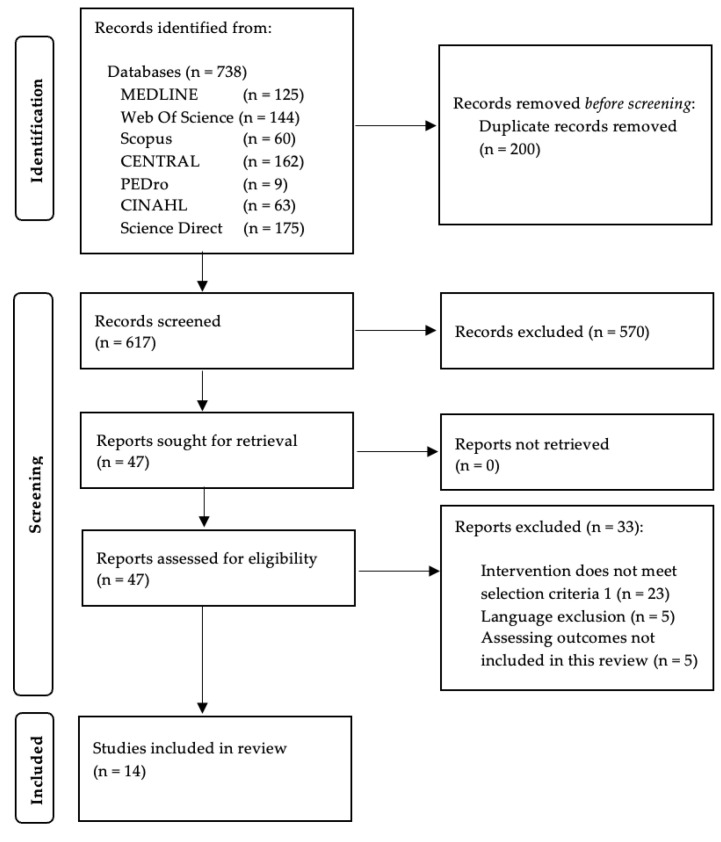
PRISMA flow diagram.

The search strategy yielded a total of 664 citations. After excluding the articles not meeting the inclusion criteria and after including supplementary articles by cross-references, a total of 14 studies were considered for the final analysis. Figure 1 displays the flowchart of the search strategy.

### 3.2. Characteristics of the Included Studies

A total of 340 healthy older adults were included (mean age 69.42 years, 220 women and 120 men) (Table 1). Eight studies examined the effects of LL-BFR training versus traditional HL resistance training [28,50,51,52,53,54,55,56], and seven studies evaluated the effects of LL-BFR training versus traditional LL resistance training [28,57,58,59,60,61,62]. The LL-BFR training workload was set at 20–40% 1RM, whereas the traditional HL resistance training workload was 70–80% 1RM, and the traditional LL resistance training workload was 20–30% 1RM. The prescribed cuff pressures varied widely, ranging from 67 mm Hg [56] to 270 mm Hg [60,61]. Seven studies employed cuff pressures greater than the limb occlusion pressure [28,50,53,54,55,60,61], and eight studies employed cuff pressures near the limb occlusion pressures [28,50,51,53,56,57,60,61]. Only Letieri et al. [28] included two BFR groups, one employing high cuff pressures and the other employing low cuff pressures. Most of the program lasted 12 weeks [50,51,54,55,56,60,61]; the shorter interventions were 4 weeks long [53,58,59] and the longest lasted 16 weeks [28]. The training was implemented two [50,51,52,54,55,60,61,62] and four [56] times per week. Eight studies included lower limb training [28,50,51,54,55,56,58,62], three studies included upper limb training [53,60,61], and three studies included upper and lower limb training [52,57,59] (Table 1). None of the studies reported serious adverse events.

### 3.3. Methodological Quality and Risk of Bias of the Included Studies

The mean PEDro score of the included studies was 5.6 (range 4–7) (Table 2). The level of inter-evaluator agreement was high for the inter-rater reliability (к = 0.86).

The risk of bias assessment of the included trials is summarized in File S2. In general, the risk of bias of the included trials in the current meta-analysis was low. The highest risk of bias was found in the reporting bias and adequate stopping rules. However, 11 studies had an unclear risk of concealment of random allocation and the blinding of the outcome assessors. Due to the nature of the groups selected for this meta-analysis, all the participants underwent prescribed physical exercise (LL, HL, or LL-BFR resistance training); therefore, we judged all the studies to be at low risk of performance bias. The totality of the assessed studies adhered to strict protocols, a necessary requirement in exercise interventions in order to reduce the risk of differential behavior by the personnel delivering the intervention [63].

### 3.4. Low-Load Blood Flow Restriction Training versus High-Load Resistance Training

**Figure 2:**
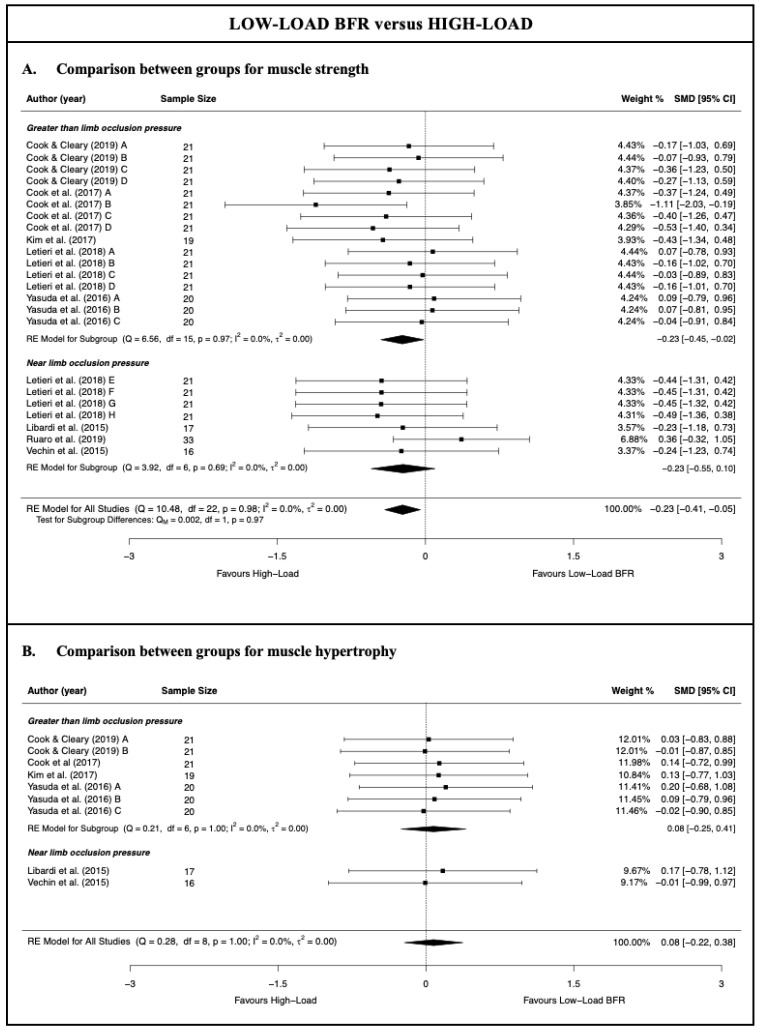
Synthesis forest plot for muscle strength and hypertrophy for low-load blood flow restriction training versus high-load resistance training [28,50,51,52,53,54,55,56].

There was moderate-quality evidence from 8 studies [28,50,51,52,53,54,55,56] (23 comparisons; n = 189) that HL resistance training produces a small and statistically significant increase in muscle strength compared with LL-BFR training (SMD = −0.23 [−0.41; −0.05]; Z = −2.52; *p* = 0.012) (Figure 2 and Table 3). There was low-quality evidence from six studies [50,51,53,54,55,56] (9 comparisons; n = 114), given that there was no statistically significant difference between the two types of training for the muscle hypertrophy (SMD = 0.08 [−0.22; 0.38]; Z = 0.52; *p* = 0.60) (Figure 2 and Table 3). The heterogeneity was not significant, with an I^2^ of 0% for both the muscle strength and hypertrophy analysis (strength: Q = 10.48; *p* = 0.98; hypertrophy: Q = 0.28; *p* = 1.00). For both the muscle strength and hypertrophy analyses, no single study significantly affected the overall SMD, and no evidence of publication bias was detected (symmetrical shape of the funnel plot; Egger’s test, *p* > 0.05) (Files S3 and S4).

With respect to the subgroup meta-analysis according to the degree of limb cuff pressure applied to perform the training, the results showed no statistically significant differences in terms of the muscle strength between the overall SMD obtained by applying pressure greater than or near that which was required to occlude the limb and that obtained with the HL resistance training (*p* = 0.97). In contrast to the meta-analysis results from the subgroup with that greater than the limb occlusion pressure, the subgroup with the near limb occlusion pressure showed a similar increase in muscle strength to that obtained with HL resistance training (moderate-quality evidence; four studies [28,51,52,56] and seven comparisons; n = 87; SMD = −0.23 [−0.55; 0.10]; Z = −1.37; *p* = 0.17). The Ruaro et al. [52] study likely had a strong influence on the results of the meta-analysis; however, that study could be considered as an outlier. In fact, the leave-one-out analysis suggested that when removing the Ruaro et al. [52] study, HL resistance training produced a greater increase in muscle strength than LL-BFR training at the near limb occlusion pressure.

For the muscle hypertrophy, a subgroup meta-analysis could be performed only for the studies which applied that greater than the limb occlusion pressure, given that only two studies/comparisons applied a near limb occlusion pressure. The results of the meta-analysis for this subgroup were very similar to those of the meta-analysis for all the studies, reporting no statistically significant differences between the application of HL resistance training and LL-BFR resistance training for the muscle hypertrophy (low-quality evidence; four studies [50,53,54,55] and seven comparisons; n = 81; SMD = 0.08 [−0.25; 0.41]; Z = 0.46; *p* = 0.65). No single study significantly affected the overall SMD obtained for this subgroup.

### 3.5. Low-Load Blood Flow Restriction versus Traditional Low-Load Resistance Training

**Figure 3:**
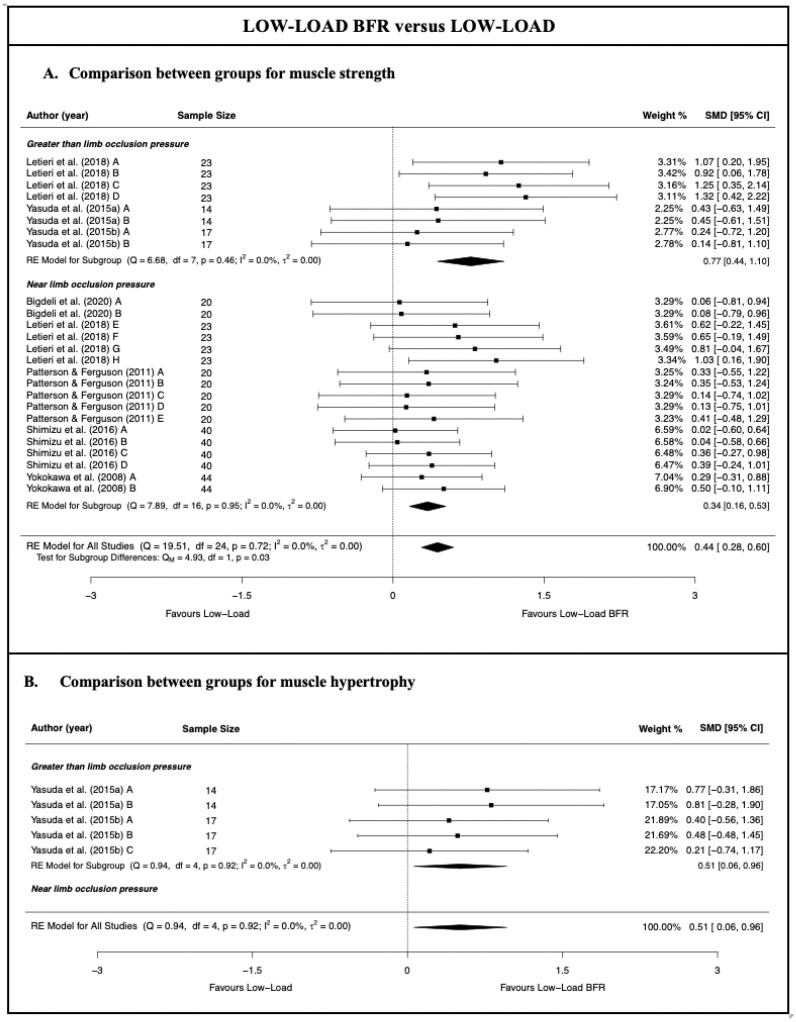
Synthesis forest plot for muscle strength and hypertrophy for low-load blood flow restriction training versus traditional low-load resistance training [28,57,58,59,60,61,62].

The meta-analysis results showed that LL-BFR training produces a small–moderate and statistically significant increase in the muscle strength (moderate-quality evidence; 7 studies [28,57,58,59,60,61,62] and 25 comparisons; n = 201; SMD = 0.44 [0.28; 0.60]; Z = 5.45; *p* < 0.001) and a moderate and statistically significant increase in muscle hypertrophy (low-quality evidence; two studies [60,61] and five comparisons; n = 31; SMD = 0.51 [0.06; 0.96]; Z = 2.22; *p* = 0.026) compared with traditional LL resistance training (Figure 3 and Table 3). The heterogeneity was not significant, with an I^2^ of 0% for both the muscle strength and hypertrophy analysis (strength: Q = 19.51; *p* = 0.72; hypertrophy: Q = 0.94; *p* = 0.92). For both the muscle strength and hypertrophy analyses, no outlier studies and no evidence of publication bias were detected (symmetrical shape of the funnel plot; Egger’s test, *p* > 0.05) (Files S5 and S6). However, eliminating the Yasuda et al. 2015a [60] study would imply an absence of statistically significant differences in muscle hypertrophy between the two training modalities.

For muscle strength, the results of the subgroup meta-analysis showed that, compared with traditional LL resistance training, LL-BFR training applied with a greater than limb occlusion pressure produced a greater increase in muscle strength than LL-BFR training applied with near limb occlusion pressure (*p* = 0.026). Although there is moderate-quality evidence that both LL-BFR subgroups obtained statistically significant differences compared with traditional LL resistance training, LL-BFR applied with a greater than limb occlusion pressure obtained a large overall effect size (three studies [28,60,61] and eight comparisons; n = 54; SMD = 0.77 [0.44; 1.10]; Z = 4.57; *p* < 0.001), whereas LL-BFR applied with the near limb occlusion pressure obtained a small overall effect size (5 studies [28,57,58,59,62] and 17 comparisons; n = 147; SMD = 0.34 [0.16; 0.53]; Z = 3.72; *p* < 0.001). No single study significantly affected the overall SMD obtained for these subgroups.

No subgroup meta-analysis by the degree of the limb cuff pressure could be performed for muscle hypertrophy, given that no study had applied near limb occlusion pressure.

## 4. Discussion

This is the first study to systematically assess the effect of LL-BFR resistance training compared with traditional resistance training on the muscle strength and hypertrophy in a healthy population older than 60 years, achieving the lowest heterogeneity compared with previous reviews [17,18,19] and allowing for a better interpretation of the results. The review provided moderate-quality evidence suggesting that, compared with traditional HL resistance training, LL-BFR resistance training promotes lower muscle strength gains, as well as low-quality evidence showing similar improvements in muscle hypertrophy in older adults. However, LL-BFR resistance training offered greater increases in terms of muscle strength (moderate-quality evidence) and hypertrophy (low-quality evidence) than traditional LL resistance training in this population. This study is also a pioneering review of the influence of BFR cuff pressure on muscle strength and hypertrophy in adults older than 60 years. There was no difference in either the muscle strength or hypertrophy when applying a pressure greater than or near the pressure required to occlude the limb blood flow compared with traditional HL resistance training. Nevertheless, greater improvements occurred in the muscle strength when higher cuff pressures were applied versus traditional LL resistance training.

The pooled data suggest that traditional HL resistance training is more effective than LL-BFR resistance training for increasing the muscle’s strength but not the hypertrophy (although it is correlated, an increased muscle strength is not always reflected in an increased muscle size [64]). However, the low-effect sizes observed are still too small to make this assertion with certainty. These results are consistent with those found in healthy younger and older adults [17,18,65]. HL resistance training has been reported to cause greater mechanical strain on the trained musculature than LL-BFR resistance training [66]. This increased strain can occur by mechanotransduction, enhancing the release of localized hormones [67] and the recruitment of fast twitch fibers [68], mechanisms that promote increased muscle strength levels. In healthy young adults, the strength gains attributable to neural adaptations are approximately 60% [69]; in older adults, however, the contribution of neural adaptations is even greater [70]. Studies have reported that there is greater electromyographic activity in HL resistance training (approximately 80% of maximum voluntary isometric contraction [MVIC]) compared with LL-BFR resistance training (approximately 30% MVIC) [68,71], inducing neuronal plasticity that leads to a greater recruitment of motor units in the medium term and thus a greater adaptation in terms of muscle strength.

Based on the results of this review, LL-BFR resistance training offers superior increases in the muscle strength and hypertrophy than traditional LL resistance training. The effect sizes observed indicate a greater robustness in the results. These findings agree with those in the literature regarding older adults [17,18], younger adults [65], and those with musculoskeletal injuries [72]. Metabolic stress appears to play a major role in the physical effects of LL-BFR resistance training, given that a similar mechanical strain has been suggested between traditional LL resistance training and LL-BFR resistance training [66,68]. In contrast to traditional LL resistance training, there is a lower oxygen bioavailability during BFR training [73], which accelerates the onset of anaerobic glycolysis in muscles [21], and consequently the accumulation of fatigue metabolites such as lactate [21,59,73,74] and hydrogen ions [21]. These mechanisms correlate with local hormone secretion [62,73,74], cell swelling [75], muscle damage [76], and the increased production of reactive oxygen species [77], all of which are related to the anabolic muscle phenomena.

Our study’s second aim was to determine the effect of LL resistance training with the effect that varying degrees of BFR cuff pressure has on the muscle strength and hypertrophy in this population. Not even the high pressures applied to the cuff during LL-BFR resistance training mimicked the benefits of HL resistance training, suggesting that the additional metabolic stress of BFR is not comparable to the mechanical and metabolic stress provided by HL resistance training. However, when comparing LL-BFR resistance training versus traditional LL resistance training, higher muscle strength increases were observed when higher cuff pressures were applied. These findings differ from those obtained in a previous meta-analysis, which indicated that the cuff pressure in BFR resistance training did not influence muscle strength and hypertrophy in older adults [18]. Nevertheless, these results should be interpreted with caution, given that the review included studies with individuals younger than 60 years with various conditions that could influence the results. In addition, that meta-analysis considered the cuff pressure value in mmHg, whereas our meta-analysis dichotomized this variable according to whether the cuff pressure was greater than the limb occlusion pressure. Using the absolute value of the cuff pressure may be slightly more inaccurate, as the pressure required to occlude a limb depends on other variables such as the limb circumference. Hence, it might be better to analyze whether a pressure greater than the limb occlusion pressure was applied to the limb than the absolute value. Physiologically, a higher restriction pressure could imply a lower blood supply and consequently higher metabolic stress during exercise, which could cause a greater increase in muscle strength and hypertrophy. This hypothesis is supported by the results of the study by Letieri et al. [28] with two groups that performed BFR resistance training using high (185.75 ± 5.45 mm Hg) and low (105.45 ± 6.5 mm Hg) cuff pressures with the same methodology. The authors observed greater increases in the muscle strength and hypertrophy in the high-pressure group after 6 weeks of training. In contrast, other studies have observed no differences between the groups with different cuff pressures in only one session [78,79]. The discrepancy between the results could partly be due to the duration of the interventions. Therefore, there is a need to investigate the middle-term effects of the cuff pressure on muscle strength and hypertrophy.

Based on the results of this review, the pressure applied to the cuff during LL-BFR resistance training could affect exercise adaptations. However, given that the differences are small and based on few studies, these conclusions have been interpreted with caution. In fact, the Ruaro et al. [52] study could be considered to be an outlier among studies employing pressures near the limb occlusion pressure because BFR resistance training was only applied to a wrist flexion exercise, which was a movement not included in the HL resistance training group. Given that no improvements were expected in the HL resistance training group, the benefits of the LL-BFR intervention were therefore exaggerated. The leave-one-out analysis suggested that, when removing the Ruaro et al. [52] study, HL resistance training produced a greater increase in muscle strength than LL-BFR resistance training with a near limb occlusion pressure. More research is needed to investigate the clinical and physiological effects of high or low pressures applied to the cuff during LL-BFR resistance training.

Within the clinical implications of this review, the most effective intervention to counteract the progressive physiological deterioration that occurs with age appears to be HL resistance training. However, the use of LL-BFR resistance training could represent an alternative training method for individuals intolerant to higher-intensity training protocols and in cases where its application is contraindicated. Thus, for healthy adults older than 60 years without contraindications, LL-BFR resistance training may be prescribed in combination with HL resistance training to aim for optimal muscular strength and hypertrophy responses. It is well known that LL-BFR resistance training is more uncomfortable than traditional LL resistance training [57,80]. Therefore, motivated older adults could start with LL-BFR resistance training while the less motivated could start with traditional LL resistance training and progressively integrate BFR resistance training. After adapting to the cuff, higher pressures could be introduced, given that higher pressures are presumed to be more effective in improving muscle strength in older adults.

This systematic review and meta-analysis presents certain limitations. The training protocols used in the included studies are widely heterogeneous in terms of their duration, type of exercise (i.e., static or dynamic contractions), and BFR application parameters. Optimal protocols for BFR resistance training are still to be established and are needed to standardize the trial interventions. The studies included in certain sub-analyses are scarce, which could lead to possible changes in the conclusions if new studies are added. No subgroup meta-analysis according to the degree of the limb cuff pressure could be performed for muscle hypertrophy. The small sample size and the high risk of bias in the treatment effect estimates for the selected studies could also have influenced the results of this systematic review. More clinical trials with a high methodological quality are therefore needed to overcome these limitations.

## 5. Conclusions

There was moderate-quality evidence that, compared with HL resistance training, LL-BFR resistance training promotes lower muscle strength gains but similar improvements in the muscle hypertrophy (low-quality evidence) in a healthy population older than 60 years. The benefits in terms of the muscle strength (moderate-quality evidence) and hypertrophy (low-quality evidence) were greater for LL-BFR resistance training than with traditional LL resistance training in older adults. There was no difference in either the muscle strength or hypertrophy when applying pressure greater than or near the pressure required to occlude the limb blood flow compared with HL resistance training. However, it appears that applying cuff pressures above the limb occlusion pressure could enhance increases in the muscle strength compared with traditional LL resistance training (moderate-quality evidence). In this systematic review and meta-analysis update, we found evidence of an increased interest in the efficacy of LL-BFR resistance training on the muscle strength and hypertrophy in healthy older adults; however, the certainty of the evidence using the GRADE methodology is still low to moderate.

## Figures and Tables

**Table 1:** Methodological characteristics and results of included studies.

Author, Year and Design	Sample Characteristics	Intervention Groups	Resistance Training Protocol	Outcomes	Results
Bigdeli et al., 2020 [57] RCT	N = 20 Age: 67.7 ± 5.8 years	*LL-BFR training* (25–35% 1RM) (N = 10) - Pressure: 50–70% AOP (~138 mmHg for lower limb; ~70.6 mmHg for upper limb) - Cuff: 5 cm (Ghamat pooyan, Tehran, Iran) *LL resistance training* (25–35% 1RM) (N = 10)	*Exercise mode* 11 functional upper and lower limb exercises *Volume* (set × rep) 2–4 × 10 *Frequency* 6 weeks; 3 days/week	*Assessments* Pre–post training *Muscle strength* 1RM knee extension/chest press	*Muscle strength* No differences were observed in muscle strength between LL-BFR training and LL resistance training at 6 weeks.
Cook et al., 2017 [54] RCT	N = 24 Age: 75.6 years, 95% CI: [73.4–78.5]	*LL-BFR training* (30% 1RM) (N = 12) - Pressure: 184 ± 25 mm Hg - Cuff: 6 × 83 cm (D.E. Hokanson, Inc., Bellevue, WA, USA) *HL resistance training* (70% 1RM) (N = 12)	*Exercise mode* Leg extension, leg curl, leg press *Volume* (set × rep) 1–3 × failure *Frequency* 12 weeks: 2 days/week	*Assessments* Pre-6 weeks-post training *Muscle strength* 1RM leg extension/leg curl/leg press MVIC knee extension (60°) *Muscle hypertrophy* Quadriceps CSA	*Muscle strength* At 6 weeks, HL resistance training showed significant improvements in 1RM leg press and MVIC knee extension compared to LL-BFR training. At 12 weeks, HL resistance training only showed significant improvements for 1RM knee extension in favour of LL-BFR training. *Muscle hypertrophy* No significant differences were detected between LL-BFR training and HL resistance training at 12 weeks.
Cook and Cleary 2019 [55] RCT	N = 21 Age: 76.35 ± 7.78 years	*LL-BFR training* (30% 1RM) (N = 10) - Pressure: 184 ± 25 mm Hg - Cuff: 6 × 83 cm (D.E. Hokanson, Inc., Bellevue, WA, USA) *HL resistance training* (70% 1RM) (N = 11)	*Exercise mode* Leg flexion and extension, leg curl *Volume* (set × rep) 1–3 × failure *Frequency* 12 weeks: 2 days/week	*Assessments* Pre–post training *Muscle strength* 10RM knee flexion/extension MVIC knee flexion/extension *Muscle hypertrophy* Quadriceps CSA Hamstrings CSA	*Muscle strength* LL-BFR training only obtained significant improvements in 10RM knee extension compared to HL resistance training. *Muscle hypertrophy* No significant differences were observed in muscle CSA between groups at 12 weeks.
Kim et al., 2017 [53] RCT	N = 19 Age: 62.74 ± 1.01 years	*LL-BFR training* (20% MVC) (N = 9) - Pressure: ~160 mm Hg - Cuff: 16 × 30 cm (D.E. Hokanson, Inc., Bellevue, WA, USA) *HL resistance training* (75% MVC) (N = 10)	*Exercise mode* Isometric handgrip contractions *Volume* (set × rep) 3 × failure *Frequency* 4 weeks; 3 days/week	*Assessments* Pre–post training *Muscle strength* MVIC handgrip *Muscle hypertrophy* Forearm girth	*Muscle strength* No significant differences were observed in muscle strength between LL-BFR training and HL resistance training at 4 weeks. *Muscle hypertrophy* No significant differences were observed in muscle hypertrophy between LL-BFR training and HL resistance training at 4 weeks.
Letieri et al., 2018 [28] RCT	N = 44 Age: 68.8 ± 5.09 years	*LL-BFR training high pressure* (20–30% 1RM) (N = 11) - Pressure: 185.75 ± 5.45 mm Hg - Cuff: Not stated *LL-BFR training low pressure* (20–30% 1RM (N = 11) - Pressure: 105.45 ± 6.5 mm Hg - Cuff: Not stated *HL resistance training* (70–80% 1RM) (N = 10) *LL resistance training* (20–30% 1RM) (N = 12)	*Exercise mode* Squat, leg press, knee extension and leg curl *Volume* (set × rep) LL-BFR: 3–4 × 15–30 LL: 3–4 × 15–30 HL: 3–4 × 6–8 *Frequency* 16 weeks; 3 days/week	*Assessments* Pre–post training and 6 weeks detraining *Muscle strength* Isokinetic peak torque of knee flexion/extension (60°/seg)	*Muscle strength* All groups presented greater increases in isokinetic peak torque of knee flexion/extension compared to LL resistance training at 16 weeks. No significant differences were observed in muscle strength between LL-BFR training either with high or low pressure and HL resistance training at 16 weeks. There were statistically significant detriments in muscle strength in the LL-BFR training with high pressure and HL resistance training group at 6 weeks detraining.
Libardi et al., 2015 [56] RCT	N = 18 Age: 64.7 ± 4.1 years	*LL-BFR training* (20–30% 1RM) (N = 10) - Pressure: 67 ± 8.0 mm Hg - Cuff: 17.5 × 9.20 cm *HL resistance training* (70–80% 1RM) (N = 8)	*Exercise mode* Leg press *Volume* (set × rep) LL-BFR: 3 × 15 HL: 4 × 10 *Frequency* 12 weeks; 2 days/week	*Assessments* Pre–post training *Muscle strength* 1RM leg press *Muscle hypertrophy* Quadriceps CSA	*Muscle strength* No significant differences were observed in muscle strength between LL-BFR training and HL resistance training at 12 weeks. *Muscle hypertrophy* No significant differences were observed in muscle hypertrophy between LL-BFR training and HL resistance training at 12 weeks.
Patterson and Ferguson 2011 [58] Crossover trial	N = 10 Age: 67 ± 3 years	*LL-BFR training* (25% 1RM) (N = 10) - Pressure: 110 mm Hg - Cuff: Not stated (CC17RB and SC10RB, Hokanson) *LL resistance training* (25% 1RM) (N = 10)	*Exercise mode* Plantar flexion *Volume* (set × rep) 3 × failure *Frequency* 4 weeks; 3 days/week	*Assessments* Pre–post training *Muscle strength* MVIC plantar flexion (90°) Isokinetic peak torque of plantar flexion (30, 60 and 120°/s) 1RM plantar flexion	*Muscle strength* The LL-BFR leg showed a significant increase in muscle strength except for the isokinetic peak torque of plantar flexion at 60° and 120°/s.
Ruaro et al., 2019 [52] RCT	N = 33 Age: 65.94 ± 4.59 years	*LL-BFR training* (40% 1RM) (N = 16) - Pressure: 70% SBP - Cuff: Not stated *HL resistance training* (70% 1RM) (N = 17)	*Exercise mode* Wrist flexion *Volume* (set × rep) 3 × 15 *Frequency* 14 weeks; 2 days/week	*Assessments* Pre–post training *Muscle strength* 1RM wrist flexion	*Muscle strength* LL-BFR training showed a significant improvement in muscle strength compared to HL resistance training.
Shimizu et al., 2016 [59] RCT	N = 40 Age: 71 ± 4 years	*LL-BFR training* (20% 1RM) (N = 20) - Pressure: 134 ± 16 mm Hg upper limb; 163 ± 17 lower limb - Cuff: 10 and 7 cm width, lower and upper limb, respectively *LL resistance training* (20% 1RM) (N = 20)	*Exercise mode* Leg extension, leg press, rowing and chest press *Volume* (set × rep) 3 × 20 *Frequency* 4 weeks; 3 days/week	*Assessments* Pre–post training *Muscle strength* 1RM leg extension/leg press/rowing/chest press	*Muscle strength* No significant differences were observed in muscle strength between LL-BFR training and LL resistance training at 4 weeks.
Vechin et al., 2015 [51] RCT	N = 16 Age: 64.04 ± 3.81years	*LL-BFR training* (20–30% 1RM) (N = 8) - Pressure: 71 ± 9 mm Hg - Cuff: 18 cm wide *HL resistance training* (70–80% 1RM) (N = 8)	*Exercise mode* Leg press *Volume* (set × rep) LL-BFR: 4 × 30–15 HL: 4 × 10 *Frequency* 12 weeks; 2 days/week	*Assessments* Pre–post training *Muscle strength* 1RM leg press *Muscle hypertrophy* Quadriceps CSA	*Muscle strength* HL resistance training obtained significant increases in 1RM leg press compared to LL-BFR training at 12 weeks. *Muscle hypertrophy* No significant differences in muscle hypertrophy between LL-BFR training and HL resistance training were observed at 12 weeks.
Yasuda et al., 2016 [50] RCT	N = 20 Age: 71.06 ± 6.61 years	*LL-BFR training* (35–45% 1RM) (N = 10) - Pressure: 120–200 mm Hg - Cuff: 5 cm width (KAATSU Master, KAATSU Japan Co., Ltd., Tokyo, Japan) *HL resistance training* (70–90% 1RM) (N = 10)	*Exercise mode* Squat and knee extension with elastic bands *Volume* (set × rep) LL-BFR: 3 × 30–15 HL: 3 × 12–13 *Frequency* 12 weeks; 2 days/week	*Assessments* Pre–post training *Muscle strength* 1RM leg press/knee extension MVIC knee flexion/extension (80°) *Muscle hypertrophy* Quadriceps/adductors/hamstrings/gluteus maximus CSA Mid-thigh and lower leg girth	*Muscle strength* There were only significant increases in the LL-BFR training regarding the MVIC knee extension compared to the HL resistance training at 12 weeks. *Muscle hypertrophy* There were only significant increases in the LL-BFR training regarding the quadriceps CSA compared to the HL resistance training at 12 weeks.
Yasuda et al., 2015a [60] RCT	N = 14 Age: 69.5 ± 7.06 years	*LL-BFR training* (26–30% 1RM) (N = 7) - Pressure: 120–270 mm Hg - Cuff: 3 cm width (KAATSU Master, KAATSU Japan Co., Ltd., Tokyo, Japan) *LL resistance training* (28–30% 1RM) (N = 7)	*Exercise mode* Arm curl and triceps press down with elastic bands *Volume* (set × rep) LL-BFR: 4 × 15–30 LL: 4 × 15–30 *Frequency* 12 weeks; 2 days/week	*Assessments* Pre–post training and 12-week detraining *Muscle strength* MVIC elbow flexion/extension (90°) *Muscle hypertrophy* Elbow flexors/extensors CSA	*Muscle strength* LL-BFR training showed significant increase in MVIC elbow extension, but not in flexion compared to LL resistance training at 12 weeks. No detraining appears in the MVIC elbow extension at 12 weeks post-intervention. *Muscle hypertrophy* LL-BFR training showed a significant improvement in both CSA outcomes compared to LL resistance training. No detraining appears in the elbow flexors CSA at 12 weeks post-intervention.
Yasuda et al., 2015b [61] RCT	N = 17 Age: 70.12 ± 5.95 years	*LL-BFR training* (26–30% 1RM) (N = 9) - Pressure: 120–270 mm Hg - Cuff: 3 cm width (KAATSU Master, KAATSU Japan Co., Ltd., Tokyo, Japan) *LL resistance training* (28–30% 1RM) (N = 8)	*Exercise mode* Arm curl and triceps press down with elastic bands *Volume* (set × rep) LL-BFR: 4 × 15–30 LL: 4 × 15–30 *Frequency* 12 weeks; 2 days/week	*Assessments* Pre–post training *Muscle strength* MVIC elbow flexion/extension (90°) *Muscle hypertrophy* Elbow flexors/extensors CSA - Upper arm girth	*Muscle strength* LL-BFR training showed a significant improvement in muscle strength compared to LL resistance training. *Muscle hypertrophy* LL-BFR training showed a significant improvement in both muscle hypertrophy outcomes compared to LL resistance training.
Yokokawa et al., 2008 [62] RCT	N = 44 Age: ≥65 years (~71.61 ± 4.34 years)	*LL-BFR training* (N = 19) - Pressure: 70–150 mmHg - Cuff: 3.3 × 14 cm *LL resistance training* (N = 25)	*Exercise mode* Half squats, forward lunges, calf raises, knee lifts, crunches, knee flexion and extension Exercises to enhance posture and dynamic stability *Volume* 90 min/week *Frequency* LL-BFR: 8 weeks; 2 days/week LL: 8 weeks; 1 days/week	*Assessments* Pre–post training *Muscle strength* MVIC knee extension (90°)/handgrip	*Muscle strength* No significant differences were observed between LL-BFR training and LL resistance training at 8 weeks.

1RM, one repetition maximum; AOP, arterial occlusion pressure; CI, confidence interval; CSA, cross-sectional area; HL, high load; LL, low load; LL-BFR, low load blood flow restriction; MVC, maximum voluntary contraction; MVIC, maximum voluntary isometric contraction; RCT, randomized controlled trial; SBP, systolic blood pressure.

**Table 2:** PEDro scores for included studies (n = 14).

Study	Random Allocation	Concealed Allocation	Groups Similar at Baseline	Participant Blinding	Therapist Blinding	Assessor Blinding	<15% Dropouts	Intention- to-Treat Analysis	Intergroup Difference Reported	Point Estimate and Variability Reported	Total
Bigdeli et al., 2020 [57]	Y	N	Y	N	N	Y	Y	Y	Y	Y	7
Cook and Cleary 2019 [55]	Y	N	Y	N	N	N	Y	Y	Y	Y	6
Cook et al., 2017 [54]	Y	N	Y	N	N	Y	Y	N	Y	Y	6
Kim et al., 2017 [53]	Y	N	Y	N	N	N	N	N	Y	Y	4
Letieri et al., 2018 [28]	Y	N	Y	N	N	Y	Y	N	Y	Y	6
Libardi et al., 2015 [56]	Y	N	Y	N	N	N	Y	Y	Y	Y	6
Patterson and Ferguson 2011 [58]	N	N	Y	N	N	N	Y	Y	Y	Y	5
Ruaro et al., 2019 [52]	Y	Y	Y	N	N	N	N	N	Y	Y	5
Shimizu et al., 2016 [59]	Y	N	Y	N	N	N	Y	Y	Y	Y	6
Vechin et al., 2015 [51]	Y	N	Y	N	N	N	N	N	Y	Y	4
Yasuda et al., 2016 [50]	Y	N	Y	N	N	N	Y	Y	Y	Y	6
Yasuda et al., 2015a [60]	Y	N	Y	N	N	N	Y	Y	Y	Y	6
Yasuda et al., 2015b [61]	Y	N	Y	N	N	N	Y	Y	Y	Y	6
Yokokawa et al., 2008 [62]	Y	N	Y	N	N	N	Y	N	Y	Y	5
N = No, Y = Yes								Mean			5.6

**Table 3:** GRADE evidence profile for the effects of low-load blood flow restriction training.

Outcome Comparison; Number of Studies (Comparisons); Sample Size		Risk of Bias	Inconsistency	Indirectness of Evidence	Imprecision	Publication Bias	SMD (95% CI)	Certainty of Evidence
*Muscle strength*								
LL-BFR vs. HL (overall effect); 8 studies (23 comparisons); n = 189		Not serious	Not serious	Not serious	Serious ^b^	No	−0.23 (−0.41 to −0.05) *	⨁⨁⨁◯ MODERATE
Greater than limb OP; 5 studies (16 comparisons); n = 102		Not serious	Not serious	Not serious	Serious ^b^	No	−0.23 (−0.45 to −0.02) *	⨁⨁⨁◯ MODERATE
Near limb OP; 4 studies (7 comparisons); n = 87		Not serious	Not serious	Not serious	Serious ^b^	No	−0.23 (−0.55 to 0.10)	⨁⨁⨁◯ MODERATE
LL-BFR vs. LL (overall effect); 7 studies (25 comparisons); n = 201		Not serious	Not serious	Not serious	Serious ^b^	No	0.44 (0.28 to 0.60) *	⨁⨁⨁◯ MODERATE
Greater than limb OP; 3 studies (8 comparisons); n = 54		Not serious	Not serious	Not serious	Serious ^b^	No	0.77 (0.44 to 1.10) *	⨁⨁⨁◯ MODERATE
Near limb OP; 5 studies (17 comparisons); n = 147		Not serious	Not serious	Not serious	Serious ^b^	No	0.34 (0.16 to 0.53) *	⨁⨁⨁◯ MODERATE
*Muscle hypertrophy*								
LL-BFR vs. HL (overall effect); 6 studies (9 comparisons); n = 114		Serious ^a^	Not serious	Not serious	Serious ^b^	No	0.08 (−0.22 to 0.38)	⨁⨁◯◯ LOW
Greater than limb OP; 4 studies (7 comparisons); n = 81		Serious ^a^	Not serious	Not serious	Serious ^b^	No	0.08 (−0.25 to 0.41)	⨁⨁◯◯ LOW
Near limb OP; 2 studies (2 comparisons); n = 33		—	—	—	—	—	—	—
LL-BFR vs. LL (overall effect); 2 studies (5 comparisons); n = 31		Serious ^a^	Not serious	Not serious	Serious ^b^	No	0.51 (0.06 to 0.96) *	⨁⨁◯◯ LOW
Greater than limb OP; 2 studies (5 comparisons); n = 31		Serious ^a^	Not serious	Not serious	Serious ^b^	No	0.51 (0.06 to 0.96) *	⨁⨁◯◯ LOW
Near limb OP;	—	—	—	—	—	—	—	—

* Statistically significant differences; ^a^ more than 50% of the studies/comparisons presented a risk of bias for attrition; ^b^ sample size fewer than 400 patients; LL-BFR, low-load blood flow restriction; HL, high-load; LL, low-load; OP, occlusion.

## Data Availability

Not applicable.

## Supplementary Materials

The following supporting information can be downloaded at: https://www.mdpi.com/article/10.3390/jcm11247389/s1, File S1: Search strategy; File S2: Risk of bias summary and graph; File S3: Sensitivity and Publication Bias Funnel Plots for the Comparison in Muscle strength be-tween Low-Load Blood Flow Restriction Resistance Training and High-Load Resistance Training; File S4: Sensitivity and Publication Bias Funnel Plots for the Comparison in Muscle Hypertrophy between Low-Load Blood Flow Restriction Resistance Training and High-Load Resistance Training; File S5: Sensitivity and Publication Bias Funnel Plots for the Comparison in Muscle Strength be-tween Low-Load Blood Flow Restriction Resistance Training and Traditional Low-Load Resistance Training; File S6: Sensitivity and Publication Bias Funnel Plots for the Comparison in Muscle Hypertrophy between Low-Load Blood Flow Restriction Resistance Training and Traditional Low-Load Resistance Training.
