# Supplementary material for: Effects of Low-Load Blood Flow Restriction Resistance Training on Muscle Strength and Hypertrophy Compared with Traditional Resistance Training in Healthy Adults Older Than 60 Years: Systematic Review and Meta-Analysis
Source: J Clin Med. 2022 Dec 13;11(24):7389. doi: 10.3390/jcm11247389 (PMC9787798; doi:10.3390/jcm11247389)
Supplement: Supplementary file 1 [file jcm-11-07389-s001.zip › File S1. Search strategy.pdf]

File S1. Full search strategy

MEDLINE

1. elderly [Title/Abstract] OR "elderly people" [Title/Abstract] OR “Elder”
2. older [Title/Abstract] OR “older adults” [Title/Abstract] OR “old people” [Title/Abstract]
3. aged [Title/Abstract] OR aging [Title/Abstract] OR ageing [Title/Abstract]
4. senior [Title/Abstract] OR geriatric [Title/Abstract]
5. #1 OR #2 OR #3 OR #4
6. "blood flow restriction therapy" [Title/Abstract] OR "blood flow restricted" [Title/Abstract] OR "blood flow-restricted" [Title/Abstract] OR "restricted leg blood flow" [Title/Abstract] OR “restriction training” [Title/Abstract]
7. "vascular occlusion" OR “KAATSU”
8. #6 OR #7
9. ((clinical[Title/Abstract] AND trial[Title/Abstract]) OR clinical trials as topic[MeSH Terms] OR clinical trial[Publication Type] OR random*[Title/Abstract] OR random allocation[MeSH Terms] OR therapeutic use[MeSH Subheading])
10. #5 AND #8 AND #9

WEB OF SCIENCE

1. elderly OR aged OR older OR geriatric OR old people OR old people
2. blood flow restriction training OR blood flow restricted OR bfrt OR KAATSU
3. muscle strength OR isometric OR isokinetic OR 1RM OR 1 repetition maximum OR hypertrophy OR muscle cross-sectional area OR muscle size OR muscle growth

4. randomized controlled trial OR controlled clinical trial OR random OR randomly OR trial
5. #1 AND #2 AND #3 AND #4

Scopus

1. elderly OR aged OR older OR geriatric OR elderly AND people OR old [Article title, abstract, keywords]
2. blood AND flow AND restriction AND training OR blood AND flow AND restriction AND therapy OR blood AND flow AND restricted OR restriction AND training OR occlusion AND training OR vascular AND occlusion OR bfrt OR KAATSU [Article title, abstract, keywords]
3. #1 AND #2

Cochrane Controlled Register of Trials (CENTRAL)

4. "blood flow restriction" OR "blood flow restricted" OR "restriction training" OR KAATSU [Title, abstract, keyword]
5. elderl* OR old* OR "older adults" OR age* [Title, abstract, keyword]
6. "muscle strength" OR "muscle force" OR "physical function" OR isometric OR isokinetic OR "1RM" OR "1 repetition maximum" OR torque OR hypertrophy OR "muscle cross-sectional area" OR "muscle size" OR "muscle growth" [Title, abstract, keyword]

PEDro

Abstract & Title: blood flow restrict* restriction training

Therapy: strength training

Problem: no selection

Body part: no selection

Subdiscipline: Gerontology

Topic: No selection

Method: Clinical trial

When Searching: AND

CINAHL

1. elderly OR aged OR older OR elder OR geriatric OR elderly people OR old people OR old people OR senior
2. blood flow restriction training OR blood flow restriction therapy OR blood flow restricted OR restriction training OR occlusion training OR vascular occlusion OR bfrt OR KAATSU
3. muscle strength OR muscle force OR physical function OR isometric OR isokinetic OR 1RM OR 1 repetition maximum OR torque OR hypertrophy OR muscle cross-sectional area OR muscle size OR muscle growth

Science direct

1. "blood flow restriction training" OR "blood flow restriction therapy" OR "restriction training" OR "occlusion training" OR KAATSU
2. elderly OR aged OR older OR geriatric
3. #1 AND #2
