# Supplementary material for: Effects of Low-Load Blood Flow Restriction Resistance Training on Muscle Strength and Hypertrophy Compared with Traditional Resistance Training in Healthy Adults Older Than 60 Years: Systematic Review and Meta-Analysis
Source: J Clin Med. 2022 Dec 13;11(24):7389. doi: 10.3390/jcm11247389 (PMC9787798; doi:10.3390/jcm11247389)
Supplement: Supplementary file 1 [file jcm-11-07389-s001.zip › File S2. Risk of bias summary and graph.pdf]

File S2. Risk of bias summary and graph.

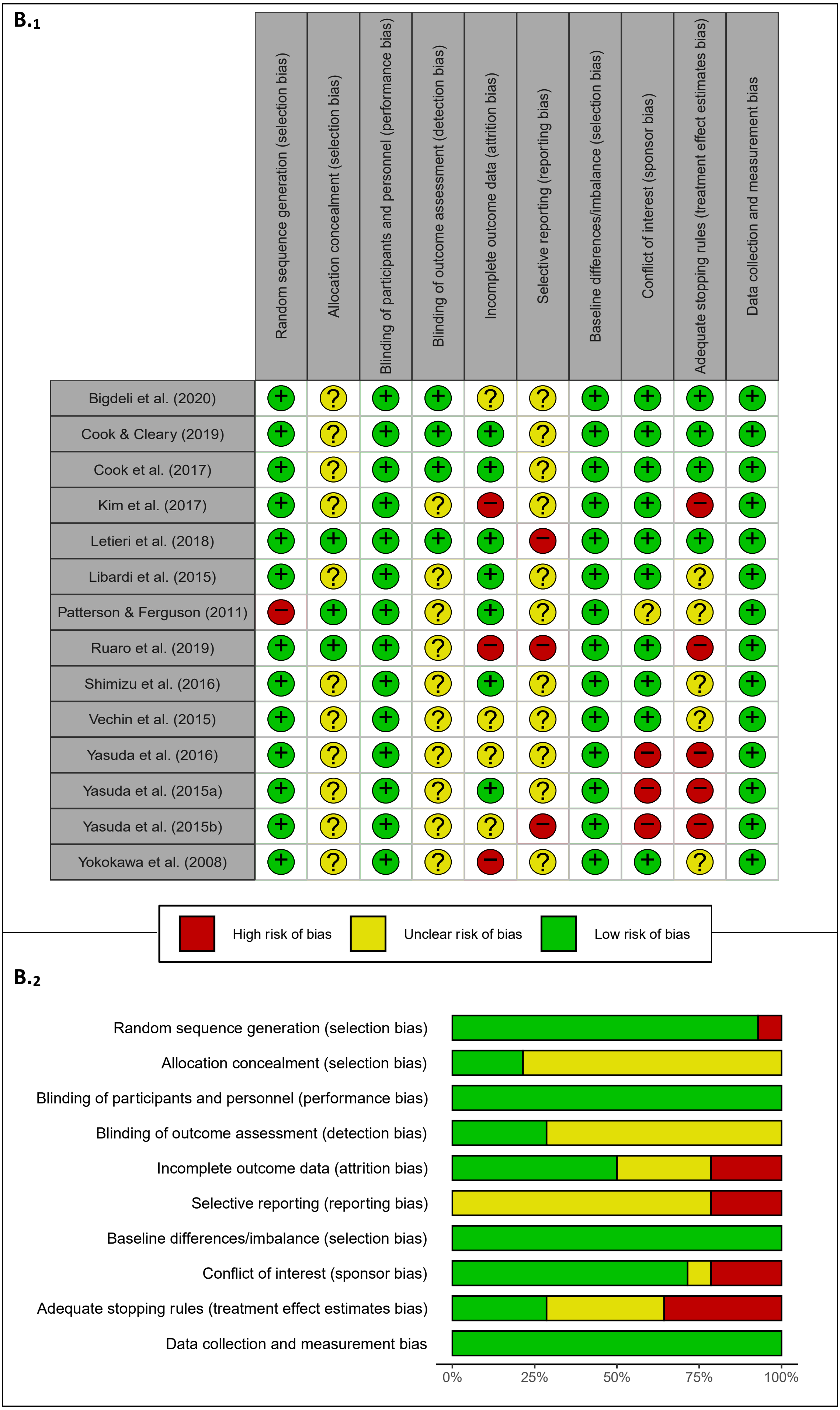

B.1 Risk of bias summary: review authors' judgements about each Risk of bias item for each included study.

B.2 Risk of bias graph: review authors' judgements about each Risk of bias item presented as percentages across all included studies.
