# Supplementary material for: Effects of Low-Load Blood Flow Restriction Resistance Training on Muscle Strength and Hypertrophy Compared with Traditional Resistance Training in Healthy Adults Older Than 60 Years: Systematic Review and Meta-Analysis
Source: J Clin Med. 2022 Dec 13;11(24):7389. doi: 10.3390/jcm11247389 (PMC9787798; doi:10.3390/jcm11247389)

File S3. Sensitivity and publication bias funnel plots for the comparison in muscle strength between low-load blood flow restriction training and high-load resistance training.

Overall effect

Leave-one-out sensitivity analysis

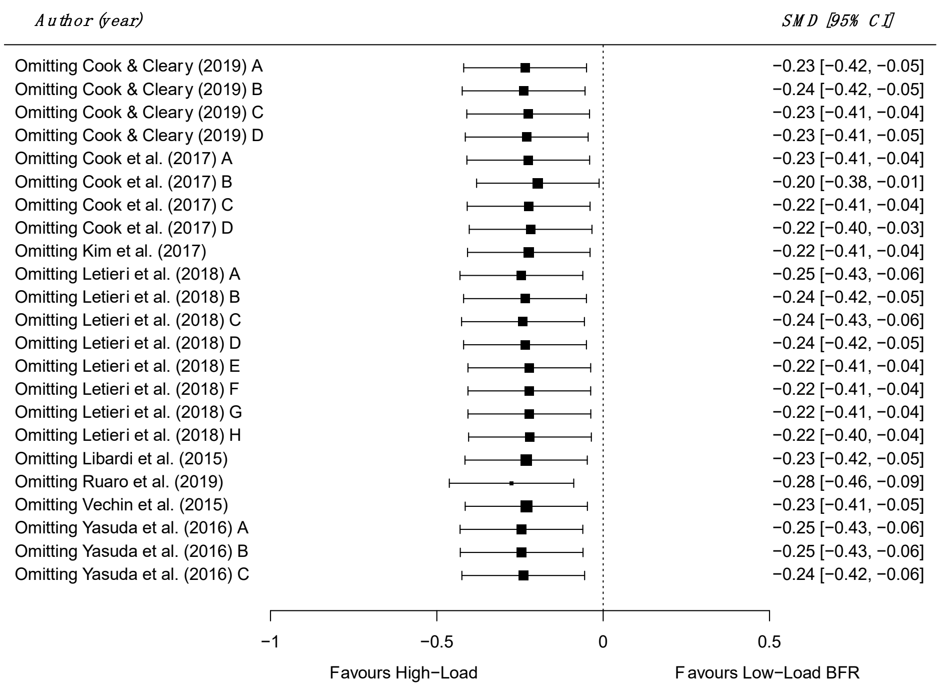

Publication bias

Egger's regression test:
Estimate: -5.95; Z = -1.85; *p*=0.064

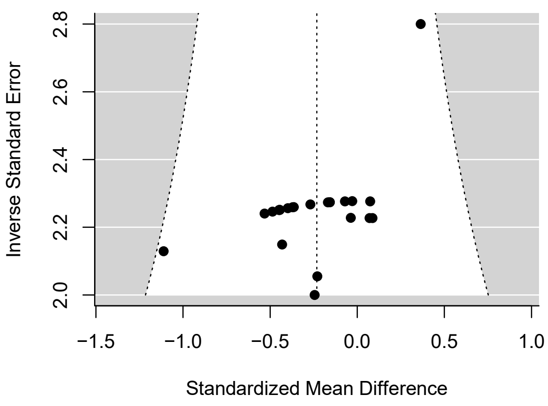

Greater than limb occlusion pressure

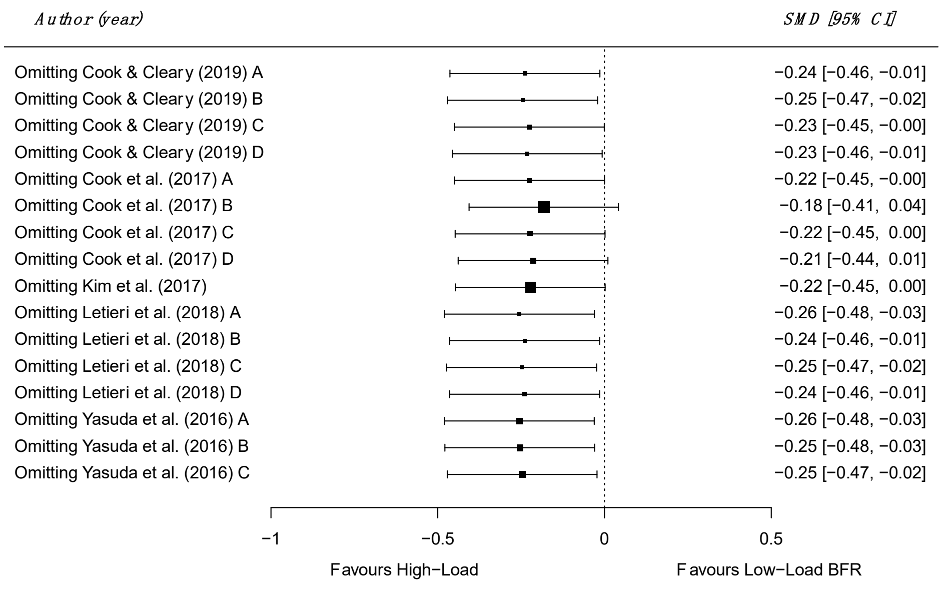

Egger's regression test:
Estimate: -19.35; Z = -1.59; *p*=0.111

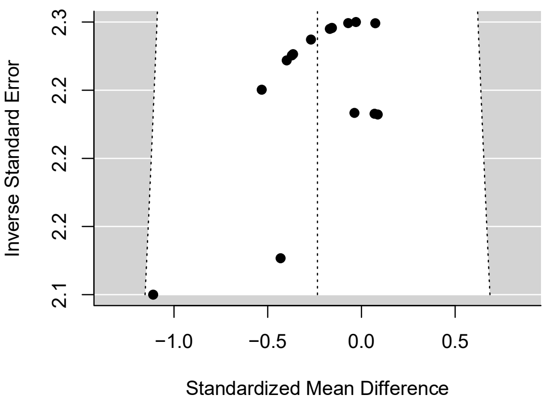

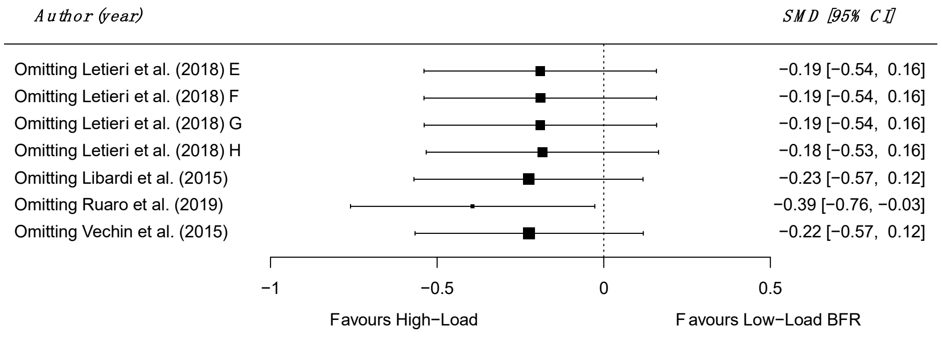

Egger's regression test:
Estimate: -5.06; Z = -1.50; *p*=0.134

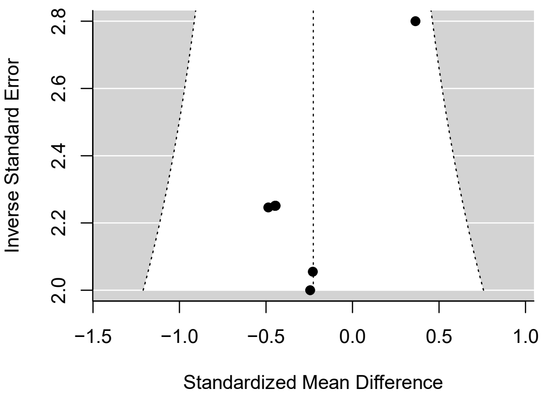

Supplement: Supplementary file 1 [file jcm-11-07389-s001.zip › File S3. Sensitivity strength High-Load.pdf]
