# Supplementary material for: Effects of Low-Load Blood Flow Restriction Resistance Training on Muscle Strength and Hypertrophy Compared with Traditional Resistance Training in Healthy Adults Older Than 60 Years: Systematic Review and Meta-Analysis
Source: J Clin Med. 2022 Dec 13;11(24):7389. doi: 10.3390/jcm11247389 (PMC9787798; doi:10.3390/jcm11247389)

File S4. Sensitivity and publication bias funnel plots for the comparison in muscle hypertrophy between low-load blood flow restriction training and high-load resistance training.

Overall effect

Leave-one-out sensitivity analysis

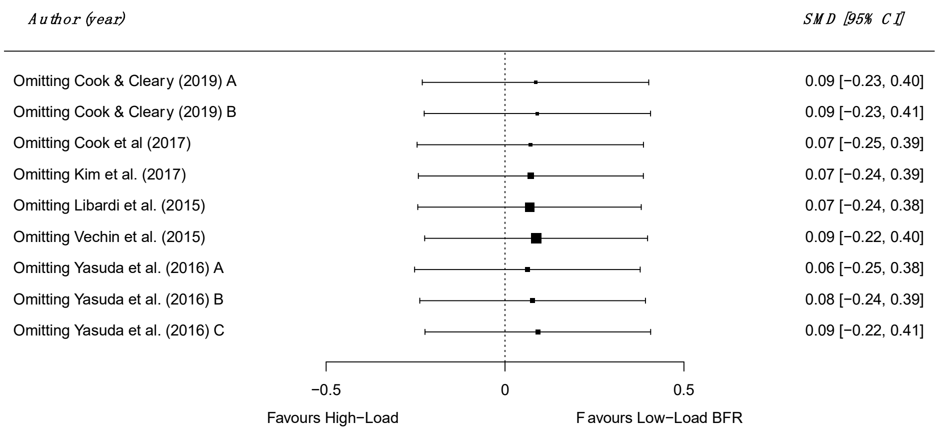

Publication bias

Egger's regression test:
Estimate: 0.31; Z = 0.04; p=0.967

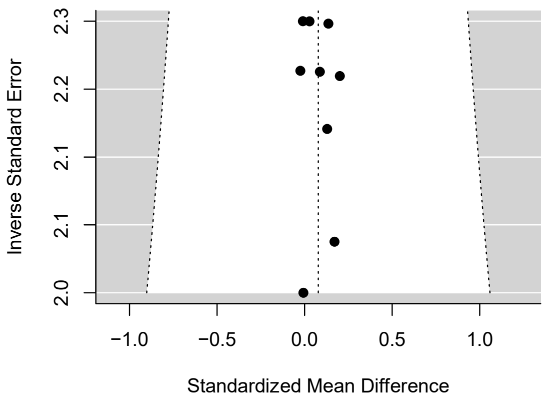

Greater than limb occlusion pressure

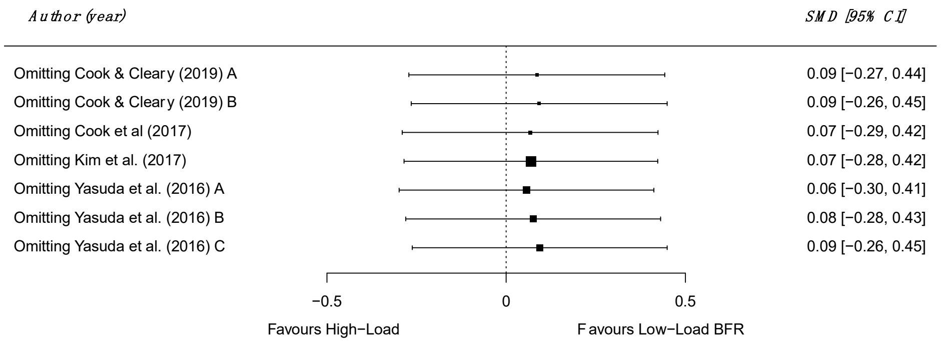

Egger's regression test:
Estimate: 3.80; Z = 0.18; p=0.860

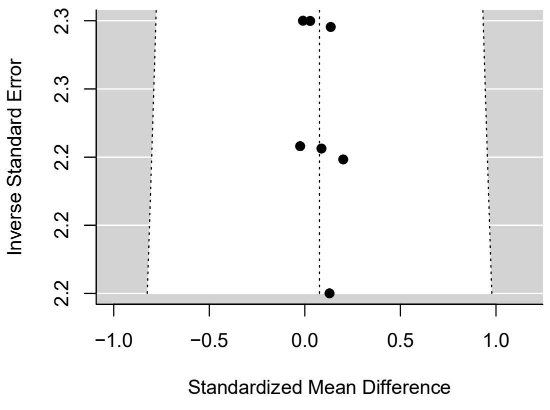

Supplement: Supplementary file 1 [file jcm-11-07389-s001.zip › File S4. Sensitivity hypertrophy High-Load.pdf]
