# Supplementary material for: Effects of Low-Load Blood Flow Restriction Resistance Training on Muscle Strength and Hypertrophy Compared with Traditional Resistance Training in Healthy Adults Older Than 60 Years: Systematic Review and Meta-Analysis
Source: J Clin Med. 2022 Dec 13;11(24):7389. doi: 10.3390/jcm11247389 (PMC9787798; doi:10.3390/jcm11247389)

File S6. Sensitivity and publication bias funnel plots for the comparison in muscle hypertrophy between low-load blood flow restriction training and low-load resistance training.

Overall effect

Leave-one-out sensitivity analysis	Publication bias

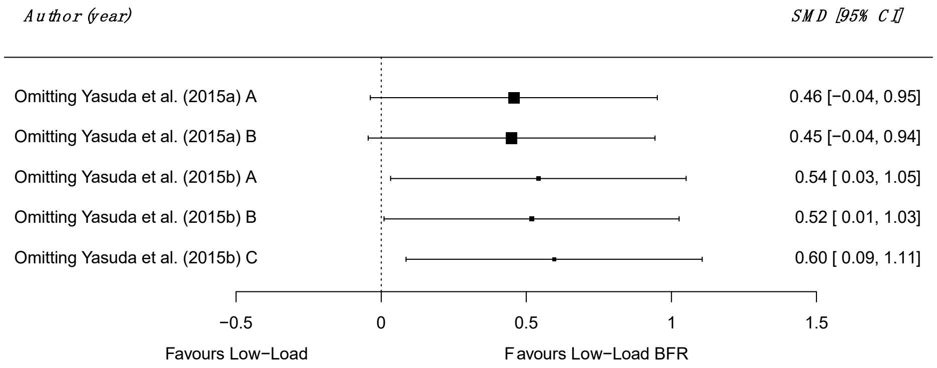

Egger's regression test:
Estimate: 6.74; Z = 0.90; p=0.367

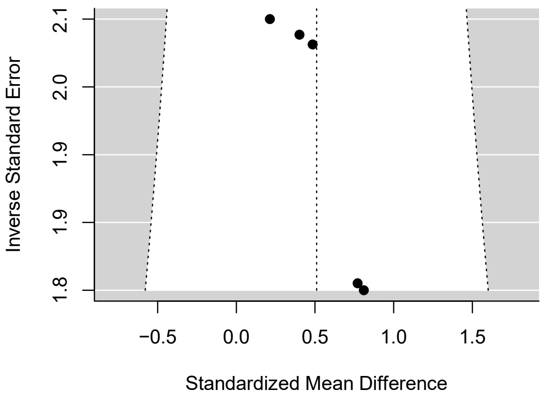

Greater than limb occlusion pressure

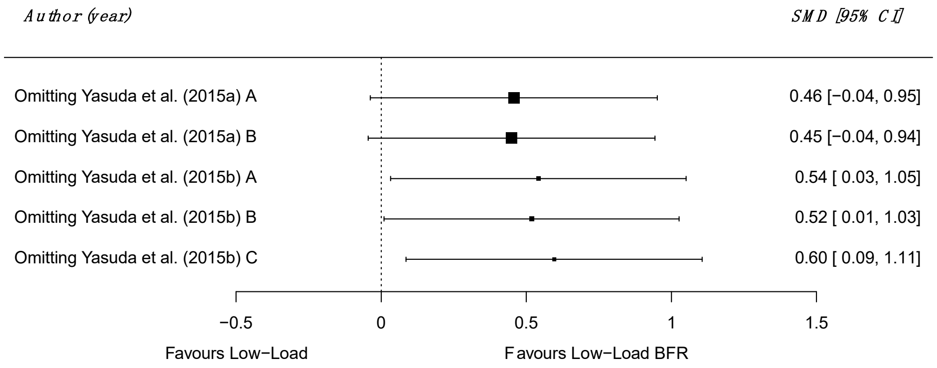

Egger's regression test:
Estimate: 6.74; Z = 0.90; p=0.367

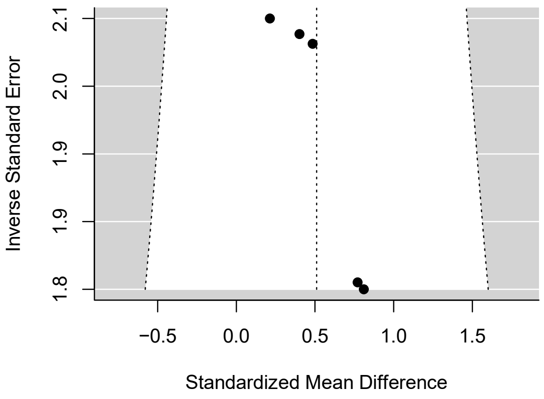

Supplement: Supplementary file 1 [file jcm-11-07389-s001.zip › File S6. Sensitivity hypertrophy Low-Load.pdf]
